# Safety and Efficacy of Loading Doses of Vitamin D: Recommendations for Effective Repletion

**DOI:** 10.3390/ph17121620

**Published:** 2024-11-30

**Authors:** Béla E. Tóth, István Takács, Kristóf Kádár, Sara Mirani, Miklós Vecsernyés, Péter Lakatos

**Affiliations:** 1 Department of Pharmaceutical Surveillance and Economy, Faculty of Pharmacy, University of Debrecen, 4032 Debrecen, Hungary; sara.mirani@pharm.unideb.hu (S.M.); vecsernyes.miklos@pharm.unideb.hu (M.V.); 2 Department of Internal Medicine and Oncology, Semmelweis University, 1085 Budapest, Hungary; takacs.istvan@semmelweis.hu (I.T.); lakatos.peter@semmelweis.hu (P.L.); 3 Department of Oral Biology, Semmelweis University, 1085 Budapest, Hungary; kadkris@outlook.hu

**Keywords:** vitamin D3, safety, deficiency, loading dose, maintenance dose, BMI, calcium, overweight, 25(OH)D, CTX

## Abstract

**Background/Objectives:** Epidemiological data on vitamin D status revealed that, despite various dosage and durations of supplementation, the effectiveness often fails to achieve optimal outcomes. The need for higher doses than previously recommended was suggested, but several modifying factors should be considered, including the level of deficiency, and BMI. The objectives of this post hoc evaluation are to characterize treatment effectiveness based on the applied dose, duration and BMI; and to assess the safety aspects associated with rapid repletion of vitamin D. **Methods:** Vitamin D deficient subjects selected in the post-hoc analysis: seventy patients included from a combined loading-maintenance supplementation (300,000 IU followed by 60,000 IU) protocol and 62 deficient subjects who received a low-dose maintenance (1000 IU/day) therapy. The risk of overload and the incidence of hypercalciuria and hypercalcemia resulting from loading or post-loading maintenance were investigated. **Results:** The moderate–fast-loading schedule of 60,000 IU per week for 5 weeks, effectively achieves the target in 25(OH)D levels over 30 ng/mL for all deficient subjects, regardless of their BMI. Slower loading with lower weekly doses confirms the safety of supplementation, but the effectiveness is dependent on the subjects’ BMI; overweight and obese patients require higher doses to reach the same vitamin D levels. No difference in safety parameters observed compared to low-dose therapies. **Conclusions:** The loading treatment involving a total dose of 300,000 IU administered over 5 or 10 weeks is effective for repletion, does not lead to 25(OH)D overload, and poses no additional risks of hypercalcemia or hypercalciuria. Furthermore, there are no safety concerns regarding changes in bone resorption markers. A combination of the loading treatment with a subsequent maintenance dose of 2000 IU daily is adequate to achieve the target vitamin D levels.

## 1. Introduction

Despite a consensus among experts that dietary intake of vitamin D is insufficient to meet biologically required levels, and that the solar UVB-exposure produced endogen vitamin D, except during the summer period, is not sufficient for the general population, the supplementation needs for most individuals are significantly underestimated.

The widespread occurrence of vitamin D insufficiency (defined as a 25(OH)D level of 20–29 ng/mL) and deficiency (25(OH)D < 20 ng/mL) is more than a public health concern—it represents an unmet medical need that raises several health concerns. Current RDA recommendations for cholecalciferol at 600–800 IU per day [1] are insufficient for most people to reach adequate levels. Recent studies have highlighted that, despite supplementation, target 25(OH)D levels are often not achieved, leaving vitamin D deficiency prevalent in a significant portion of the population [2,3,4]. Trial results and observational studies suggest that the optimal endocrine/skeletal outcomes are associated with a 25(OH)D blood concentration > 30 ng/mL, without a concerning upper safety limit, as summarized by the Endocrine Society’s clinical guidelines [1]. However, some researchers have raised concerns about higher doses of vitamin D and the potential risks associated with certain treatment regimens, which may have led to dose-related adverse reactions in specific populations [2,3,4].

Vitamin D supplementation strategies have been contentious over the past two to three decades despite numerous RCTs aimed at determining the safest and most effective dosing to normalize plasma 25(OH)D concentrations. Recent studies emphasize the importance of achieving 25(OH)D concentrations > 75 nmol/L (30 ng/mL) for optimal health [1,5,6,7,8]. Clinical reports also highlight the risks associated with various vitamin D supplementation strategies in treating deficiency. While a broad range of daily cholecalciferol doses is used clinically without significant adverse effects, factors such as baseline 25(OH)D levels, dosing regimen (daily or intermittent), body weight, BMI, age, gender, and genetics can influence individual responses. A systematic review of 108 studies on vitamin D status published by Veugelers et al. (2015) concluded that the effective supplementation doses should be higher than previously suggested, since “host and environmental” factors may produce large variations for the general population. They also advocated for weight-specific dosing to better achieve beneficial outcomes with minimized overdose risks [9].

Daily dosing of vitamin D, as well as intermittent dosing schedules (weekly, bi-weekly, or monthly), has been shown to achieve similar 25(OH)D levels; the latter also results in advances due to better adherence in some populations [4,10,11,12,13]. Overall, the cumulative dose taken during a repletion period is crucial for raising blood 25(OH)D levels to the target range. Mathematical models predicting outcomes based on daily cholecalciferol intake dose (ranging from 1000 to 10,000 IU) provide reasonable good estimates of the outcome over a few weeks of administration [14].

However, alternative bolus dosing regimens—such as seasonal or yearly high doses of 300,000 to 600,000 IU—offer a more convenient supplementation option, though they raise significant safety concerns, particularly the increased risk of falls and fractures, especially among the elderly [3,15] within the first 1–3 months of treatment [2,3]. These findings prompted a reevaluation of the Tolerable Upper Intake Level for vitamin D, sparking debate over the efficacy of such therapies, and suggesting reduced upper limits for elderly populations [2]. The increased fracture risk of high-dose supplementation in vitamin D-deficient patients might be linked to transient changes in bone metabolism and elevated bone turnover markers, which could also predict fracture risk in postmenopausal women and the elderly [16].

Systematic reviews of safety studies confirm that long-term daily cholecalciferol supplementation does not statistically increase the risk of hypercalcemia or kidney stones compared to placebo. The monthly supplementation of 100,000 IU over 3 years does not increase the frequency of kidney stone events or cases of hypercalcemia. It has been suggested that hypercalciuria, rather than hypercalcemia, may be used as a more sensitive indicator of an increased risk of vitamin D overdose [17] as a result of increased intestinal calcium absorption. It can be also a result of calcium overdose in supplementation, but in that case, the incidence of such an event is not directly related to the vitamin D dose [18]. Although vitamin D has a direct mineralization effect on bone and cartilage, studies suggest that extended dosing regimens during repletion may be associated with changes in bone resorption biomarkers and consequent change in calcium homeostasis. Notably, no dose-dependent association between hypercalcemia and vitamin D supplementation has been observed in doses ranging from 400 to 4800 IU/day [19]. However, long-term maintenance doses below 2800 IU/day do not appear to increase the risk of such adverse events [17], and doses above 800 IU/day remain contentious, particularly for elderly, vitamin D-deficient populations [19,20].

The current post hoc evaluation aims to assess treatment effectiveness and the proportion of therapy failure in relation to dose, duration, BMI, and age; and to examine the comparative safety aspects of two rapid repletion methods for vitamin D deficiency (combined loading–maintenance regimens) compared with low-dose maintenance (of 1000 IU/day) supplementation as an “active control”. The analysis includes a risk assessment of hypercalciuria and hypercalcemia resulting from a 300,000 IU loading dose and the combination with post-loading maintenance, compared to continuous low-dose supplementation in a vitamin D-deficient population.

## 2. Results

### 2.1. Concerns of Treatment Effectiveness

The efficacy of vitamin D3 treatment was previously reported [12,21]. Briefly, both the 300,000 IU loading protocols were found to be safe and effective in restoring the low 25(OH)D levels. The five-week-long “moderate–faster”-loading (m.load) protocol, with a daily equivalent dose of 8571 IU, resulted in a significantly higher increase in 25(OH)D, compared to the 10-week-long “slower”-loading (s.load) protocol with a daily equivalent dose of 4286 IU/d by the end of the treatment. Factors such as age and gender did not have a significant effect, but minor differences in pre-loading values or body weight were found to be significant. The mean increase in the faster-loading group was +31.04 ± 12.95 ng/mL (CI: 26.59–35.49); while for the slower-loading group, it was +25.08 ± 10.16 ng/mL, (CI: 21.59–28.57), showing a significant difference in effectiveness (*p* = 0.044) of the same total dose due to the 5- or 10-week treatment duration. Both dosing schemes resulted in improvements in 25(OH)D levels > 30 ng/mL, consistent with findings from other studies in a recent meta-analysis by Zhuang et al. [7].

**Figure 1:**
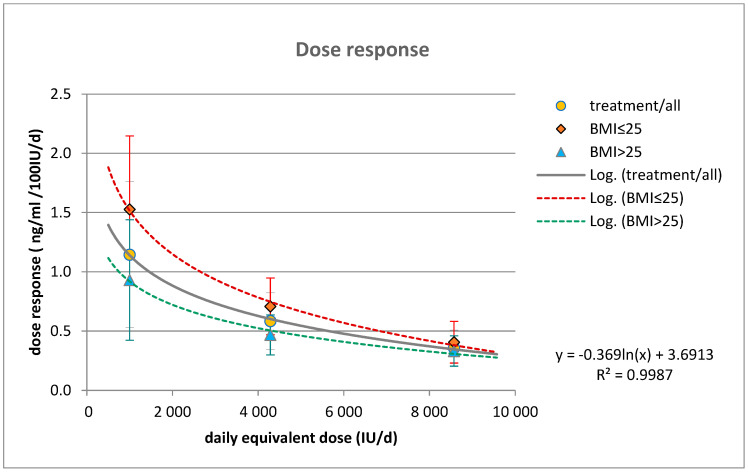
Dose response plot for the treatment groups of the two loading supplementations and the low-dose maintenance (8 weeks) calculated by the daily equivalent doses (orange circles). The red and the blue-filled symbols are indicated for the BMI ≤ 25 and BMI > 25 subgroups of treatment (means and ±SD). Logarithmic regression fitted, R^2^ = 0.998 for all of the three subsets.

The outcome of the loading schedule of 300,000 IU is at least 2–3× more powerful than the rather conservative 1000 IU/die “low-dose maintenance” (l.maint) supplementation therapy (*p* < 0.0001), which resulted in only (+11.44 ± 6.15 ng/mL; CI: 9.88–13.00) a nominal increase in 25(OH)D levels after two months of treatment, and just slightly more after the extended period to 3 months of therapy. The dose–response ratio of these three dosing arms (moderate–faster loading, slower loading, and low-dose maintenance) was 0.36/0.59/1.15 ng/mL/100 IU per day, respectively. The higher doses result in a lower proportional dose response ratio. A logarithmic regression model (y = −a × ln(dose) + b) was used to analyze the dose–response data, showing a good fit with an r^2^ = 0.9987 (Figure 1). The model suggested higher daily equivalent doses were needed for overweight–obese patients to achieve similar outcomes in 25(OH)D elevation as seen in normal-weight individuals.

**Figure 2:**
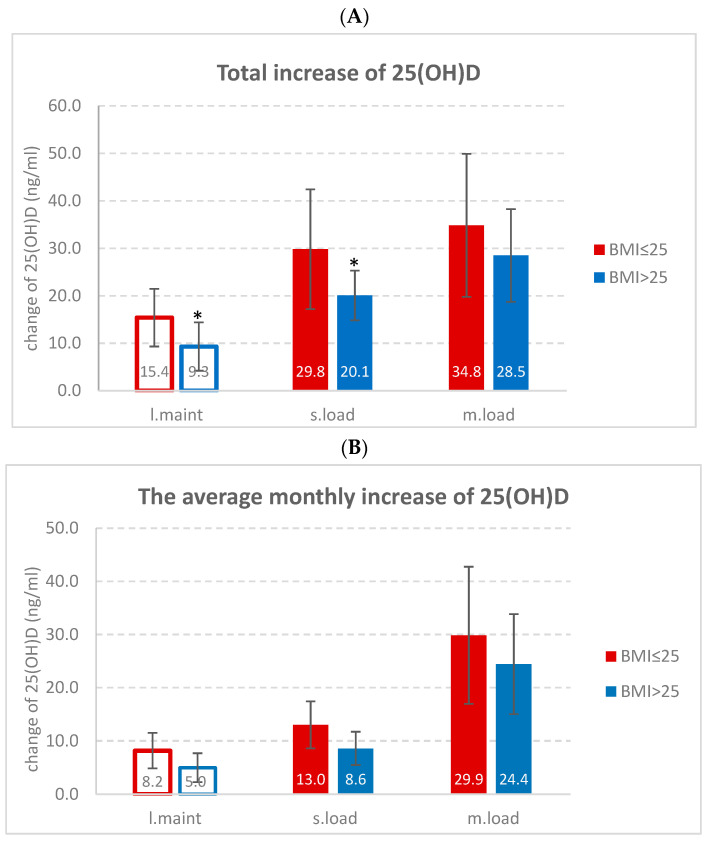
(**A**, **upper panel**): The total increase in 25(OH)D levels by the treatment: loading schedules of 10 weeks (slower loading: “s.load”) and the 5 weeks (moderate–faster loading: “m.load”) applied the similar total dose of 300,000 IU during vitamin D repletion. The low-dose maintenance (“l.maint”) of 1000 IU/d served as an active control applied in 8 weeks (marked with empty columns). The * represents the significant difference *p* < 0.001. (**B**, **lower panel**): The monthly average increase in 25(OH)D levels by the two loading schedules of 10 weeks (slower-loading) and the 5 weeks (moderate–faster loading) applied the similar total dose of 300,000 IU during vitamin D repletion. The low-dose maintenance of 1000 IU/d served as an active control applied in 8 weeks. Columns indicated mean ± SD, all values normalized to 30 treatment days.

The impact of BMI on the efficacy of loading treatments was observed as the increases in 25(OH)D levels being significantly (*p* < 0.0014) attenuated in overweight–obese subgroups in slower loading. The effect of BMI on treatment outcome was found to be non-significant for the faster-loading subgroups. (Figure 2A). Similar trends were shown also in the low-dose maintenance group, indicating a substantial difference in response based on BMI categories (25(OH)DΔ = 6.1 ng/mL, ≈40%, *p* < 0.001) (see also Table 1).

On the chart where the effectiveness was plotted by the average monthly increase (normalized for 30 days) of the treatment (Figure 2B), the difference for the 1000 IU/die low-dose maintenance was 5.0–8.3 ng/mL for the “BMI > 25” vs. the “BMI ≤ 25” subjects, compared to increase in slower loading (8.6–13.0 ng/mL) in a similar range. Rapid repletion by the moderate–faster-loading results in a prominent increase in 25(OH)D for both the high and the low BMI subgroups.

**Figure 3:**
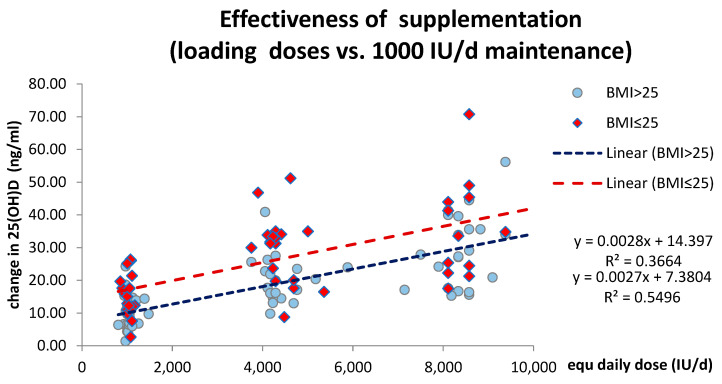
The individual changes by the response to loading doses compared to low-dose maintenance, as calculated by the daily equivalent doses (IU/d) for each trial subject. Red diamonds represent a BMI ≤ 25 and blue circle symbols represent the result of subjects with a BMI > 25. In this simplified model, the linear regression lines plotted for both the BMI ≤ 25 and BMI > 25 subgroups of treatment, which show a constant difference in intercept approx. ∆ = 7.0–8.0 ng/mL within the therapeutic range of 1000–10,000 IU/d, indicating the “relative deficit” of effectiveness for the overweight–obese subjects.

In addition to observed changes in mean 25(OH)D, the loading effectiveness is depicted by individual plots. The impact of BMI on treatment effectiveness was analyzed here using a simplified linear regression model, which showed a consistent deficit in effectiveness for overweight–obese subjects within the therapeutic range of 1000–10,000 IU/d. (Figure 3).

This simplified model for the loading calculates the dose for overweight–obese subjects DDE_(BMI>25)_ = 1.037 × X_(BMI<25)_ + 2600 (in IU/d) in order to reach a similar outcome level as a result of loading, (DDE = proposed daily dose equivalent for overweight subjects; X = dose given for normal bw. subjects) as an adjusted dosing strategy for overweight–obese patients to achieve optimal outcomes of 25(OH)D elevation.

The clinical effectiveness of treatments varies across BMI subcategories, demonstrating the highest success rate (≈100%) in achieving normalization of 25(OH)D levels above 30 ng/mL with the moderate–faster-loading regimen. In contrast, the slower-loading treatment provides only ≈80% of patient group success in reaching the target zone, with a note that one-third of the subjects form a BMI > 25 subgroup, considered as therapy failure. The low-dose maintenance therapy overall showed the lowest rates of satisfactory increase rate, particularly among overweight–obese patients. (See also Table 1).

### 2.2. No Safety Concerns Associated with the Loading Protocols

Individual-level risk assessments were conducted to evaluate the potential vitamin D overload. Obviously, no instances of overload were detected in the low-dose maintenance group. Upon loading treatments, the average 25(OH)D levels did not exceed 50 ng/mL for any of the BMI subgroups. Specifically, the confidence interval (CI) was 42.33–50.47 for BMI ≤ 25 and 36.16–42.75 ng/mL for BMI > 25. None of the individuals had serum 25(OH)D values that exceeded the safety limit of 85 ng/mL (210 nmol/L). Among the 70 subjects of loading treatments, four patients had supraoptimal 25(OH)D levels exceeding the 50 ng/mL + 20% threshold: two females (aged 64 and 28 years, one of each in the slower-load and moderate–faster-load treatment groups) and two males (aged 55 and 30 years, in the moderate–fast-loading group).

Safety assessments of these cases revealed that none of the patients with supraoptimal 25(OH)D levels exhibited abnormal serum calcium levels or any clinically significant changes in other laboratory parameters. No serious adverse events were reported within the treatment period. One patient (PAT#-718) reported four adverse events within four weeks, including palpitations, signs of hematuria, right ear pain, and subfebrile conditions with mild palpitations. Treatment with ibuprofen, diclofenac, and topical NSAIDs, as prescribed by the GP, led to the resolution of these symptoms within a few days. The safety outcomes of these cases with higher 25(OH)D results are summarized in Table 2.

### 2.3. Post-Loading Maintenance

**Figure 4:**
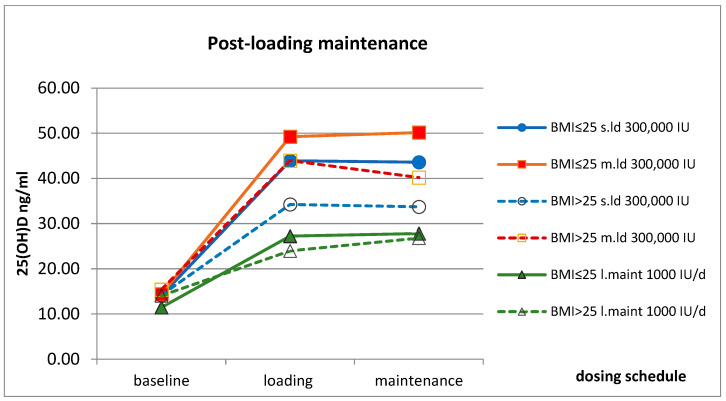
Serum 25(OH)D levels during the post-loading follow-up maintenance period by the subgroups of BMI resulted in reconciliation to steady 25(OH)D levels within the target zone. Dotted lines with open symbols indicated the subgroups of BMI > 25 patients. The two dosing schedules of loading (5 and 10 weeks) with a total dose of 300,000 IU continued with 4 weeks of the same 2000 IU/die equivalent follow-up maintenance and were compared to low-dose maintenance therapy with a continuous 1000 IU/die served as an active control (n = 62).

Post-loading maintenance was evaluated for its effectiveness in maintaining 25(OH)D levels following a vitamin D supplementation regimen. The study compared the outcomes of the post-loading maintenance dose (equivalent to 2000 IU/day, total dose of 60,000 IU) for ≈4 weeks with the continuous low-dose maintenance therapy of 1000 IU/day over a similar duration. The post-loading maintenance dose kept the mean 25(OH)D levels within the target range for the lower and higher BMI subgroups as well. Although the response to post-loading maintenance varied among individuals, the change in response to post-loading maintenance dose was non-significant in means. These variations were not specific to BMI subgroups (*p* = 0.343) or the duration of loading therapy (*p* = 0.206). (Figure 4).

**Figure 5:**
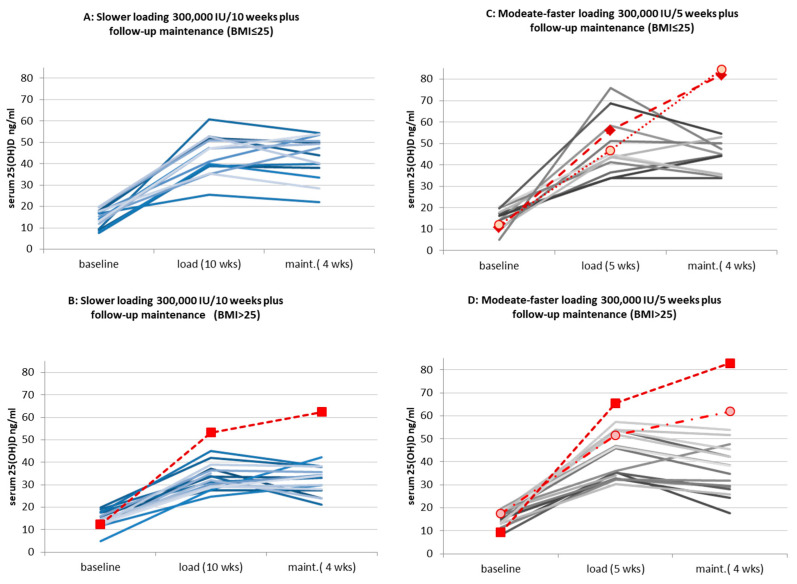
(**A**–**D**) Individual 25(OH)D levels by the end of the 4-week post-loading maintenance by subgroups in each loading treatment arm and by the BMI subgroups of patients from the ITT population. Treatment outcome of <60 ng/ml by the end of follow-up maintenance period represented with solid lines. Cases with supramaximal levels as a result of combined loading + maintenance, irrespective to protocol deviations by compliance or visit window, are highlighted with red dotted lines and various symbols.

Additionally, approximately one-third of patients experienced an unexpected significant increase in 25(OH)D levels during the follow-up period with post-loading maintenance (dose equivalent of 2000 IU/d), possibly due to delayed metabolic effects of the cholecalciferol administered during the loading period. The occurrence was similar between treatment groups (*p* = 0.44 Fischer’s exact test), but the effect was more prominent in the moderate–faster-loading treatment group, which resulted in Δ = +14.83 ± 10.77 ng/mL vs. the lower daily dose equivalent of the slower-loading group (Δ = +5.86 ± 4.88 ng/mL) (*p* = 0.021). Notably, a subset of older subjects experienced a more pronounced increase compared to younger participants during the post-loading maintenance phase, although the difference was not statistically significant. (Figure 5A–D).

For a small number of subjects, particularly those older than 45 years, the post-loading maintenance phase led to supramaximal 25(OH)D levels (>60 ng/mL), without exceeding the upper safety limit of 85 ng/mL. These cases of elevation to supramaximal level had no reported adverse events, and serum calcium and the other laboratory parameters remained in the normal range except in two cases of urinary CA/CRE elevation. (See also Table 3).

In the low-dose maintenance group, the extended dosing period with 1000 IU/day for four weeks resulted in a modest increase in 25(OH)D levels, albeit below the targeted level of 30 ng/mL. The response to this extended dosing varied with different BMIs. Overall, the effectiveness of the low-dose maintenance regimen was limited, with a substantial proportion of subjects failing to achieve optimal 25(OH)D levels even after twelve weeks, indicating incomplete supplementation and limited effectiveness of the therapy in the study.

### 2.4. Concerns Regarding Changes in Bone Metabolic Markers

As stated in our previous report, there were no changes observed in P1NP levels during the loading phase. Neither the main effect of loading (*p* = 0.142, r = 0.18) nor the main effect of the group (*p* = 0.223, r = 0.15) were found to be significant. Treatment arms were evaluated separately: P1NP levels decreased significantly in the slower-loading group (−6.64 ± 15.5, *p* = 0.03), while the moderate–faster-loading group showed a minor nonsignificant nominal increase (+1.33 ± 12.2, *p* = 0.526). There was no statistically significant difference observed between BMI groups.

No significant difference in changes resulting from loading was observed in carboxy-terminal collagen crosslinks (CTX) or in bone-specific ALP (bALP). Although individual changes resulted in a nominal decrease in means and medians of CTX for the BMI ≤ 25 subgroup compared to the BMI > 25 subgroup, these differences from baseline were non-significant in either subgroup.

**Figure 6:**
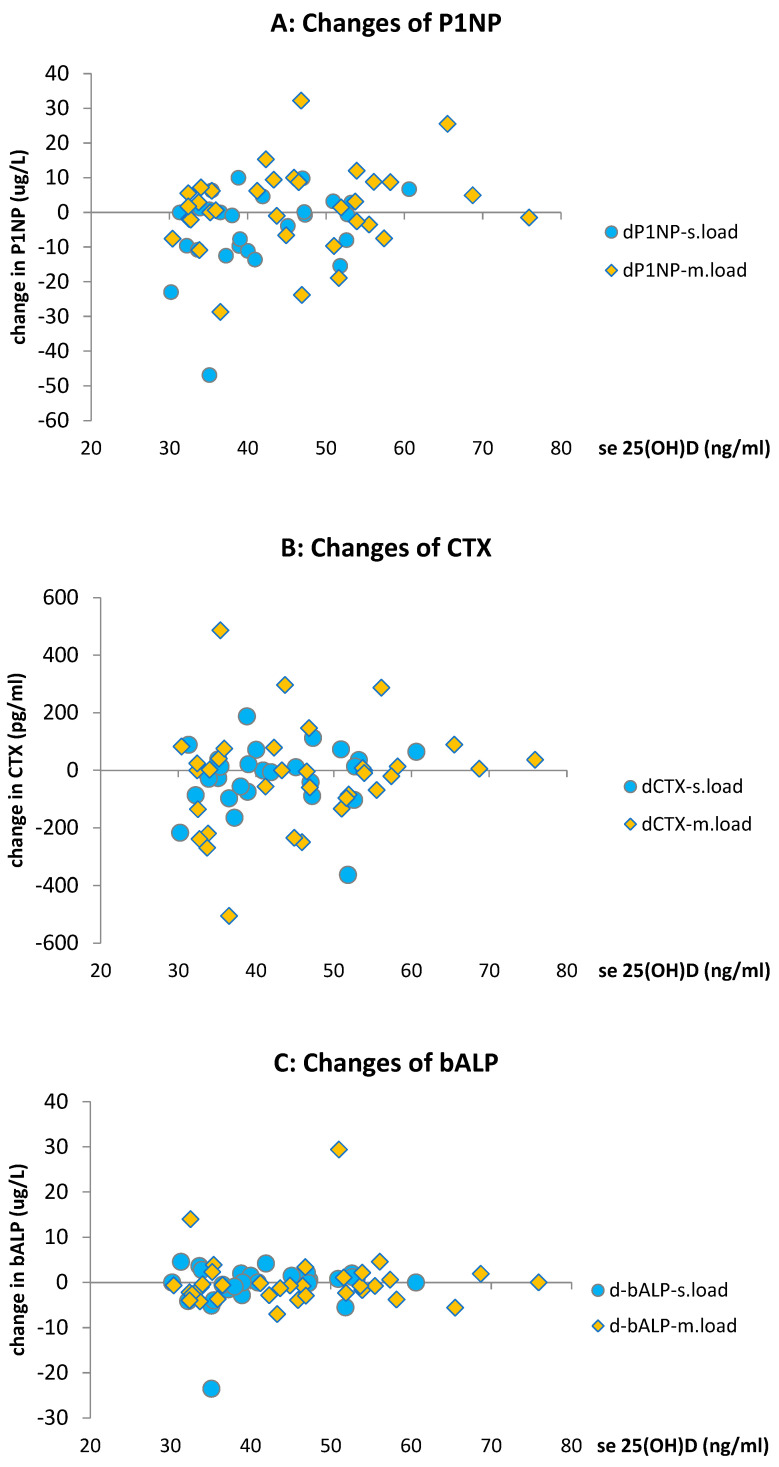
(**A**–**C**) Plots of individual changes in bone metabolic parameters (P1NP, CTX, bALP) by treatment of loading regimens (slower-loading for 10 weeks: “s.load”; and moderate–fast-loading for 5 weeks: “m.load”; both with 300,000 IU) presented by the 25(OH)D on the horizontal axis.

It is essential to note that within the dose range applied in this study (<10,000 IU/d equivalent) and with a total loading dose not exceeding 300,000 IU, there was no correlation demonstrated between the CTX parameter and post-loaded 25(OH)D levels (*p* = 0.87, r = 0.022). Taken together, the given dose range and duration of therapy applied (in 5 or 10 weeks) did not induce significant changes in bone metabolic resorption parameters. (Individual changes are presented in Figure 6A–C).

### 2.5. Concerns of Calcium Homeostasis

The changes in serum calcium and the frequency of deviations on individual levels were compared between the loading and the low-dose maintenance treatments. The 1000 IU/d therapy for 8 or 12 weeks that served as a “control group” showed no significant changes in calcium levels. Treatment with a loading dose of 300,000 IU resulted in a minor increase in serum calcium, which was marginally significant (*p* = 0.05). The changes during the follow-up maintenance period, however, were not significant. The individual changes in serum calcium levels after the loading doses were statistically different (*p* = 0.048, Mann–Whitney non-parametric test) from the low-dose maintenance therapy.

There was no correlation detected between the changes in serum calcium and the post-loaded vitamin D levels. There was no correlation detected between the post-loaded 25(OH)D levels and the changes in serum calcium (*p* = 0.574 and *p* = 0.804, for s.load and m.load doses, respectively), indicating that there is no increase in the risk of hypercalcemia by the higher-loading doses applied, or by the change in 25(OH)D (*p* = 0.572).

The risk of hypercalcemia at the individual level was assessed by the deviations of serum calcium. One case (out of 70 participants) had a transient increase in serum calcium to 2.68 mmol/L during the loading phase (with a moderate 35.4 ng/mL 25(OH)D level). A similar increase above the upper normal limit (UNL) was seen in one subject in the low-dose maintenance group. Notably, there were no signs of hypercalcemia observed in any of the subjects with supramaximal vitamin D levels.

**Figure 7:**
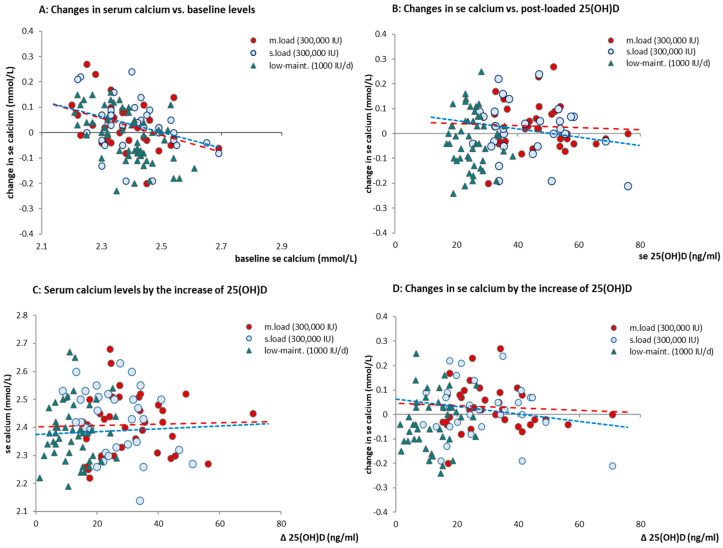
(**A**–**D**) Changes in serum calcium from baseline to post-treatment: (**A**,**B**) Changes in serum calcium by the initial calcium levels and by the post-loaded 25(OH)D levels. (**C**,**D**) Post-loaded serum calcium levels and the changes in serum calcium by the increase in Δ25(OH)D of treatment. There was no statistical correlation found between 25(OH)D and changes in serum calcium. (The trendlines, although statistically not supported, added as dashed-red lines and -blue for m.loading and s.loading, respectively. Green triangles represent the individuals of low-dose maintenance).

The individual changes in serum calcium from baseline to post-loading are depicted by the initial (baseline) serum calcium levels on the horizontal axis. There was no statistical difference in the frequency of deviations of serum calcium found when the three treatment arms were compared (*p* = 0.085), suggesting that these deviations are not dose-related. (Figure 7A,B) Due to a relatively small sample size, no statistically meaningful regression lines could be adjusted to subgroups, but the negative slope of the trendline “suggests” that the changes in the low baseline calcium levels are mostly positive, while the higher baseline calcium levels changed toward negative directions, as seen in most cases. (Figure 7C,D).

**Figure A1:**
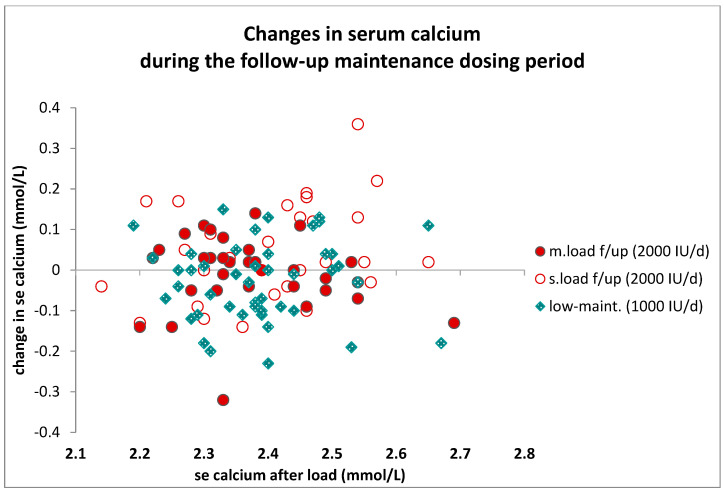
Data points of the changes of individual calcium during the post-loaded follow-up maintenance period of slower-loading (open circles) and moderate–faster-loading (filled circles), represent no statistical correlation by the post-loaded serum calcium levels. Data of extended low-dose maintenance of 1000 IU/d treatment for a duration of +30 days plotted with filled diamond symbols.

No further changes in serum calcium were observed after the loading as an effect of treatment. Post-loading maintenance therapy resulted in similar serum calcium levels in both the slower- and the moderate-loading groups, with no statistical significance. (See in Appendix A Figure A1). There was one single deviation > UNL within the post-loading follow-up period: one additional subject had increased se calcium to 2.71 mmol/L (from baseline + 6.7% increase). The occasional cases of registered higher serum calcium are presumably not closely related to changes in 25(OH)D levels and occurred in each treatment arm. These were transient in manner, and the magnitude does not possess clinical significance since serum calcium values were less than <5% above UNL.

**Figure 8:**
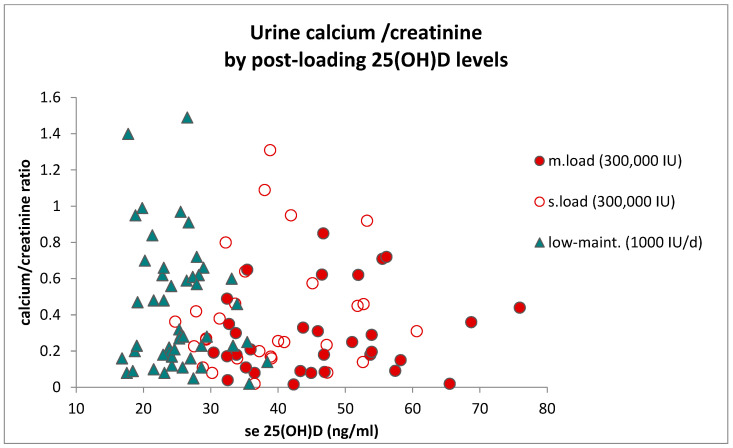
Individual urine CA/CRE ratio by the post-loading serum 25(OH)D levels. Data points of the slower-loaded (open circles) and moderate–faster-loaded (filled circles) subsets represent no correlation, i.e., no association, resulting in increments within the 20–80 ng/mL range of 25(OH)D. The CA/CRE values of low-dose maintenance of 1000 IU/d treatment for a duration of 8 weeks, presented with filled triangle symbols, are all associated with levels of 25(OH)D < 30 ng/mL.

The post hoc evaluation concerned the risk of hypercalciuria by analyzing the urinary calcium excretion based on the morning calcium/creatinine ratio (CA/CRE) by examining the changes in CA/CRE following treatment and observing the frequency of elevated values. The frequency of elevated CA/CRE cases was similar in the loading dose treatment groups (8/70; approximately 11%) compared to the low-dose maintenance group (8/62, approximately 13%). Any cases of higher CA/CRE levels detected at the beginning of the study were normalized by the end of the loading phase. Interestingly, none of the cases with supramaximal post-loading 25(OH)D levels after loading showed elevated CA/CRE levels (Figure 8).

Throughout the post-loading period, the frequency of CA/CRE deviations remained below 10%. There were no significant changes in CA/CRE values during both the loading and maintenance phases, regardless of the treatment group, BMI, age, or treatment duration. The study found no association between 25(OH)D levels and CA/CRE during the maintenance phase. Individuals with high vitamin D levels did not show increased urinary calcium levels, indicating a minimal or no risk of hypercalciuria associated with vitamin D supplementation at this observed dosing range. None of these few subjects reported adverse events possibly attributable to hypercalcemia and hypercalciuria or symptoms of cardiovascular events in our study.

Individual data points of the slower-loaded and moderate–faster-loaded subsets represented no significant association resulting in increments of CA/CRE within the 20–80 ng/mL range of 25(OH)D, which suggests that no relevant risk of hypercalciuria is associated with increased vitamin D levels as the effect of loading.

## 3. Discussion

### 3.1. Effectiveness of Loading Regimens

Effective loading dose administration protocols aim to achieve a significant increase in 25(OH)D levels in vitamin D-deficient patients within a shorter timeframe while minimizing the risk of overload or related side effects. Our study results on loading dose administration over 5 or 10 weeks showed excellent effectiveness, with a similar increase in 25(OH)D levels in adults given 5000 IU/d for 10 or more weeks, as reported by Heaney et al. [14]; in a deficient group of patients, the mean increase in 25(OH)D was around 71 nmol/L, after one month of 500,000 IU ingestion [22]. A similar total dose of 300,000 IU by a weekly 50,000 IU administration to elderly people for 6 weeks, without a baseline criterium for vitamin D deficiency, resulted in a mean of 25(OH)D concentrations 61 ng/mL, which is slightly higher than that registered in our study [11].

Considering the vitamin D-deficient population enrolled in our studies, the dose and the duration of treatment in addition to BMI were found to significantly influence efficacy, showing variances in response to the (total) dose applied. Age did not seem to significantly impact the effectiveness of loading or maintenance. This is in agreement that no significant age-related impairment in absorption or in metabolism to 25(OH)D was expected in low-dose therapies [23].

The most relevant other internal factors such as co-morbidities, endogenous glucocorticoid, genetic profile, etc., and external factors such as concomitant medications, sun exposure, and nutritional status could have influenced the effectiveness. These factors were most considered in a study in/ex criteria of enrolment. Dietary variations of daily meals, mainly the fat content and composition, could also have some but less significant impact on the absorption rate in the otherwise homogenous vitamin D-deficient study population.

It is important to note that the moderate, yet still supraphysiological, loading doses administered during the loading phase are 3 to 5 times greater than what would typically be achieved through physiological daily UVB sun exposure during photoconversion. Concerns regarding potential overload that could result in diminished effectiveness of the liver’s vitamin D-25-hydroxylase enzyme and its capacity for converting vitamin D to 25-hydroxyvitamin D have been addressed. Specifically, it has been demonstrated in earlier studies that intermittent weekly or monthly administration of moderately high doses (30,000 to 50,000 IU) yields an increase in 25(OH)D levels comparable to that achieved through cumulative daily dosing over the same period; i.e., the same effectiveness of liver conversion capacity was observed [4,10,11,12,13,24].

### 3.2. Dosing Adjusted to BMI

Given the higher prevalence of vitamin D insufficiency in individuals with a higher BMI, dose adjusting is recommended [22] by body weight or by BMI, and it is in agreement with the recommendations of guidelines [1,9,18]. The increase in vitamin D levels resulting from oral cholecalciferol supplementation, in general, is lower in obese patients, indicating a need for higher vitamin D doses to achieve complete repletion compared to normal-weight individuals [9,18,22,25].

Our post hoc analysis examined the relationship between the effectiveness of vitamin D repletion by a rapid loading therapy and subjects’ BMI, focusing on the effectiveness (as the proportion of patients that achieved the desired range), and the increment of 25(OH)D achieved as a result of loading or the post-loading maintenance doses applied. Our study results confirmed that individuals with higher BMI had a constantly diminished increase in 25(OH)D levels compared to those with a BMI < 25. A prominent difference of ≈39% was registered between the overweight and obese compared to lean subjects in the slower-loading schedule. Interestingly, less (<19%) difference was observed when the higher daily equivalent doses of loading were applied. A similar outcome was observed in obese/overweight healthy volunteer subjects in a large supplementation study. This disparity was more pronounced in overweight and obese subjects, highlighting the impact of body weight on the dose–response relationship [18].

The dose–response rate in our study was consistent with the findings from other studies [1,14,26]. For the low-dose maintenance group, it ranged from 1.541 for BMI ≤ 25 to 0.931 nmol/L per 100 IU for BMI > 25 subjects. Similarly, a slightly lower rate was observed (0.86 nmol/L per 100 IU) among overweight/obese patients [18]. Variations in dose–response rates across BMI subgroups were potentially attributed to factors such as level of depletion, size of 25(OH)D compartments, genetic variations, and lifestyle factors. Vitamin D insufficiencies in subjects exhibited negative correlations with body mass index and body fat since adipose tissue serves as a reservoir for vitamin D, and the addition of an extra amount of vitamin D was required to saturate this biological depo in obese subjects. The storage in adipose tissue has been demonstrated by radiolabeled vitamin D. The volumetric dilution hypothesis explains that oral administration of vitamin D in obese subjects stored more in adipose tissue and resulted in a lower increase in 25(OH)D in serum than that was observed in normal-weight patients [27,28].

Higher doses of vitamin D supplementation led to lower dose-response rates, with a difference between normal weight and overweight/obese individuals [26]. Our results confirmed those, since slower loading with a 4286 IU/d equivalent dose provided the range of 0.709–0.434 for normal BMI ≤ 25 and overweight–obese subjects, similar to that of 0.56 nmol/L reported for a 4000 IU/d (100 μg) dose [26]. The highest daily equivalent loading dose (8571 IU/d, ~2143 μg) in moderate–faster loading in our study provided a lower dose response with a small range of variations (0.401–0.325 nmol/L per 100 IU) in regard to low and high BMI categories, respectively.

Our results demonstrated that the faster-loading dose regimen is highly effective, as virtually all subjects (100%) achieved normalization (above 30 ng/mL) irrespective of the BMI. In contrast, the outcome of slower-loading treatment appears somewhat less impressive for patients with higher BMIs. Furthermore, the low-dose maintenance therapy provided sufficient supplementation for none (0%) of the overweight–obese vitamin D deficient patients in our study, and only a few (<20%) of the patients with a BMI ≤ 25 could attain the 30 ng/mL target zone after 2 months. Consequently, the therapy failure of a low-dose treatment is more frequent among BMI > 25 subjects, which poses a potential risk due to the lack of expected efficacy, caused by dosing inadequacy. (See also Table 4 for a summary of estimated effectiveness).

It has been concluded that BMI is a useful predictor for dose–response evaluations. In accordance with the recommendations of the Endocrine Society [1], supplementation should be 1.5 to 3 times higher for overweight or obese individuals compared to normal-weight subjects [18]. Our results, based on a simplified linear model, suggest that the adjusted daily dose equivalent (DDE) for overweight–obese patients can be calculated as follows: DDE = 1.037 × X_(dose in IU)_ + 2600 (IU) for the loading doses.

### 3.3. Effectiveness of Post-Loading Maintenance

Post-loading maintenance dosing aims to sustain optimal 25(OH)D levels post-repletion without significant declines. A combination of 500,000 IU loading plus monthly maintenance of 50,000 IU/month (≈1667 IU/die) was successful in maintaining the elevated 25(OH)D levels in deficient populations [22]. Our study demonstrated that a maintenance dose of 2000 IU/day is effective in keeping the target 25(OH)D levels in range, with some variations among individuals, but without causing a significant decline in group means.

The focus should be on two main concerns: (a) the risk of overload resulting from the high loading plus the superimposed maintenance doses, and (b) the potential risk of delayed effects of the administered doses on laboratory safety parameters. These issues were examined at the individual level within each treatment arm.

The risk of overload as a result of delayed effects of loading, in addition to maintenance doses, was maybe due to delays in transport, distribution, and metabolism of the physiological cholecalciferol conversion process. Orally ingested vitamin D travels with chylomicrons and lipoproteins, allowing for receptor-mediated, rapid hepatic delivery of vitamin D. However, it is also stored mainly in the adipose tissue as cholecalciferol [27,28] serving as a buffer for hepatic access, which could result in the superimposed production of calcidiol. Individual or altered balance between production and clearance may also contribute to a consequent rise in 25(OH)D levels. A pharmacokinetic study reported by Ilahi et al. demonstrated a rapid increase in circulating 25(OH)D levels during the first 1–2 days, followed by a delayed increase that reaches the maximum around 7–10 days after ingestion, and then a gradual descent over the next 20–50 days [29]. Similarly, another study with an intermittent administration of 50,000 IU/month doses, resulted in a delay of the increase in 25(OH)D at least by 25 days after the last dose administered, in younger participants with vitamin D deficiency and low BMI [24]. The dosing schedule we used for loading treatment arms is essentially in the similar range, attributed to a potential superimposed 25(OH)D conversion, as the physiological decline of 25(OH)D after reaching the peak is longer than the interval before the next dose is taken. The observed delays in loading reconciliation cannot be fully explained by the differences in BMI of these patients (average BMI among these subjects was 25.9 ± 5.2 kg/m^2^).

In contrast, the extended 4 weeks of follow-up dosing (with 1000 IU/d) for the low-dose maintenance group resulted in an additional increase in the group mean of 25(OH)D levels. It is important to note that the group mean of this treatment arm did not reach the target level for more than two-thirds of subjects, indicating the lower effectiveness of the low-dose maintenance, even after three continuous months of therapy.

### 3.4. Safety Concerns

Adverse events such as hypercalciuria and hypercalcemia are potentially associated with vitamin D and calcium supplementation therapy. Thus, potential vitamin D overload and early signs of hypercalcemia and hypercalciuria were investigated as therapy outcomes. The cases of slight hypercalcemia observed during the loading period or maintenance phase were low in frequency, transient, and considered clinically non-significant, with elevations of less than 2.5% above the upper normal limit. Similar occurrences were also seen in the low-dose maintenance “control” group.

Notably, the high 25(OH)D levels above 60 ng/mL in our study were not associated with hypercalcemia (or hypercalciuria) resulting from the loading doses. Similar safety results were observed with supplementation ranging from 800 to 4000 IU/d or 50,000 IU/week even with 25(OH)D levels exceeding over 80 ng/mL [11]. Consistent with findings, it was concluded that the risk of drug-related toxicity, as indicated by hypercalcemia (serum calcium > 2.7 mmol/L), does not occur at serum 25(OH)D levels below 700 nmol/L [30]. It is important to highlight that hypercalcemia was also reported in vitamin D studies with doses ranging from 400 to 800 IU/d to 4800 IU/d, plus calcium supplements up to 1200 mg daily. The study concluded that no significant association between episodes of hypercalcemia and vitamin D dose [19].

Exposure to an intermittent high-dose (100,000 IU/month) resulted in serum 25(OH)D levels exceeding 90 ng/mL in the “ViDA” study (daily equivalent dose of approximately 3300 IU/d), and had no impact on the rate of hypercalcemia within the 50–84-year-old population [17]. The results of this study showed that this bolus dosing of vitamin D increased serum 25(OH)D levels to a mean of 135 nmol/L, with a maximum observed level of 289 nmol/L, and did not result in any adverse effects related to kidney stones and hypercalcemia [17].

However, a higher level of urinary calcium excretion was reported in a study that combined low-dose vitamin D (800 IU/d) with high-dose calcium carbonate (1000 mg/d) in patients with vitamin D insufficiency, with no significant difference in hypercalcemia compared to the placebo group [31]. This finding highlights the impact of calcium intake, relative to the effect of vitamin D dose administered. Urine calcium was significantly associated with the rate of calcium absorption mediated by serum 1.25(OH)2D, as well as the level of dietary and supplemented calcium intake, but not with serum 25(OH)D.

As it was explained, 1.25(OH)2D is the active metabolite while 25(OH)D has no direct effect on intestinal calcium absorption under normal physiological conditions, unless serum levels exceed 90 ng/mL [32]. It has been suggested that patients on vitamin D and calcium supplements should be monitored for hypercalciuria. In light of the presented study results, the loading–maintenance protocols with a “moderate” total dose of 300,000 IU did not elevate the 25(OH)D above the 90 ng/mL, thereby the potential risks of hypercalciuria with controlled calcium intake were reduced [32]. Similarly, Gallagher and Smith, in their vitamin D supplementation RCT study involving women aged 57–90 years, concluded that there is no relation between hypercalcemia and the dose of vitamin D applied [19]. In our study, we also found no association between supramaximal post-loading 25(OH)D values and elevated CA/CRE levels. This suggests that loading with 300,000 IU administered over 5 or 10 weeks in weekly doses does not increase the risk of elevated urine calcium excretion compared to lower-dose vitamin D supplementation.

The effect of a long-term administration of high daily doses (4000 IU, 10,000 IU) of vitamin D, raised concerns regarding CTX levels, as there was a continuous increase, particularly prominent in the highest dose group after 18 months, while PTH decreased in the same group [15]. The change in CTX is expected to correspond with the increase in 25(OH)D over the long-term administration due to the resorptive action of vitamin D, although suppression of PTH may occur. The lower PTH reduced the effect on bone formation; thus, higher vitamin D with elevated CTX levels may modulate the osteoclast activity, potentially leading to an overall reduction in bone BMD, as observed in long-term outcomes [15].

In a shorter (8-week) supplementation study involving subjects who were not severely deficient, serum CTX exhibited a moderate positive correlation with 25(OH)D for the 4000 IU dose (which reached a mean of 40.4 ng/mL), while P1NP was not associated with 25(OH)D levels. [33]. In our study, the slower-loading treatment arms showed a decrease in P1NP levels, whereas the faster-loading group demonstrated only a minor, non-significant nominal increase after 5 weeks. Since no statistically significant difference was observed between the BMI groups, it can be concluded that the observed minor effect is likely not related to vitamin D levels, at least given the relatively short duration of the loading that we applied.

There were no significant deviations in laboratory safety parameters within the observation period of 10 to 15 weeks in our study, except for occasional cases of elevated CA/CRA, which presumably do not represent a 25(OH)D-dependent effect within the normal range of dosing. Individuals with supramaximal 25(OH)D as a result of loading did not show elevated urinary calcium levels, and only one case of deviation above the upper normal limit (UNL) was registered during the maintenance follow-up period. These findings support the view that a moderate total dose of 300,000 IU loading does not elevate 25(OH)D levels above 90 ng/mL, thereby reducing the potential risk of hypercalciuria given the otherwise controlled calcium intake.

*The possible weak points of our study are as follows:*

Our post hoc data analysis study has limitations. Firstly, the generalizability of the findings is restricted due to the small overall sample size and the specific post-hoc selection of subgroup populations within each treatment arm. Our studies only included the deficient population with a baseline assessment of blood 25(OH)D, which may not fully represent a real-world patient population.

Additionally, the analysis compared loading therapy to low-dose maintenance therapy from two separate randomized controlled trials (RCTs). While the key parameters were similar due to the enrollment criteria, this could introduce statistical bias. The small number of participants in the subgroup analysis may weaken the findings, particularly in regression analysis.

Furthermore, the study only measured bone metabolic parameters (CTX and P1NP) during the loading phase and did not extend these measurements into the follow-up/maintenance phase. There was also an imbalance in the gender distribution, which could influence outcome parameters, but was insufficient for detailed subgroup analysis. Although the gender ratio did not show significant differences in the outcomes, it remains a potential co-factor that warrants further investigation.

*The strengths of our study are as follows:*

This study is one of the first to provide a comparative efficacy and safety assessment of a rapid-loading dose schedule of 300,000 IU vs. a conservative low-dose supplementation, particularly by BMI subgroups. It found no safety concerns associated with loading doses, as evidenced by serum and urinary calcium levels, and confirmed that bone resorption markers showed no correlation between safety issues and loading dose effectiveness.

Key findings include that the rapid-loading dose regimen was highly effective, with 100% of subjects achieving normalization of vitamin D levels, regardless of BMI. The study results underline the importance of tailored dosing strategies based on BMI for optimal vitamin D repletion outcomes of loading dose schedules.

## 4. Materials and Methods

This post hoc safety and effectiveness analysis included the clinical and laboratory data of two recently reported [12,21] oral vitamin D supplementation protocols, which are intended to provide a rapid normalization of the low 25(OH)D levels. The loading–maintenance study was a randomized, controlled, multicenter, open-label study comparing two different vitamin D loading protocols of 10 weeks (named slower loading, “s.load”), and a faster loading for 5 weeks (moderate–faster loading, “m.load”), resulting in a loading dose of 300 000 IU, and followed by a maintenance dosing for additional 4 weeks (60,000 IU) for all subjects. The laboratory dataset of the low-dose maintenance supplementation (“l.maint”) schedule protocol served as an active control for safety assessments, using the oral vitamin D3 (equivalent of 1000 IU/day) for a similar duration of 8 + 4 weeks. (See also Table 5 for a summary).

Registered oral vitamin D3 film-coated tablets were used in both studies and supplied by the same Marketing Authorization Holder (MAH: Pharma Patent Ltd., Hungary). The loading protocols were supplied in both groups by the commercially available 30,000 IU vitamin D3 (cholecalciferol) oral film-coated tablets (30,000 NE Vitamin D3 Pharma Patent tablet), which were administered in different doses of 30,000 IU/week for 10 weeks for the “s.load” group and 30,000 IU tablets administered twice a week for 5 weeks for the “m.load” group. The same 30,000 IU tablets were administered during the maintenance dose follow-up period bi-weekly (for the f/up week 1 and week 3). The low-dose maintenance treatment group received either the same 30,000 IU tablets once a month or 7000 IU tablets weekly, or 1000 IU tablets daily, supplied by the same manufacturer, which resulted in supplementation equivalent to 1000 IU/day doses for all participants. There were no instructions given for tablets taken with or without food, but they were recommended to be taken at the same time of day.

Based on the efficacy and the main safety outcome parameters of these doses used for maintenance supplementation, it was confirmed that the same cumulative doses are similarly effective in various dosing regimens irrespectively of the daily, weekly, or monthly dosing intervals, thus considered as dose-equivalent [12,24]. Accordingly, all subjects who received a continuous low-maintenance dose equivalent to 1000 IU/day supplementation during the (8 + 4) weeks of their treatment period are considered as one treatment group (n = 62). Calcium citrate tablets (200 mg) were added to the diet to achieve a total calcium intake of approximately 1200 mg/day for all participants.

Subjects from the intention-to-treat (ITT) population, of ages between 18 and 82 years, with the initial serum 25-hydroxyvitamin D (25(OH)D) levels at baseline (not more than 20 ng/mL), were included in the post-hoc analysis. The main Exclusion Criteria included: vitamin D intake of more than 1000 IU per day within two months prior to screening; significant obesity (BMI > 36); chronic or serious disease, which can significantly influence the absorption and metabolism of bones or vitamin D or Ca; cardiovascular disease (included heart failure or angina pectoris); increased serum calcium level se Ca > 2.65 mmol/L results or symptoms of hypercalcemia; persistent hypercalciuria or signs of kidney stone within the last year; severe kidney disease (CKD 3 or higher); and signs or suspected alcohol or drug abuse; existence or suspected gravidity; any other finding or symptoms which may indicate a potential interference with the safety by the opinion of the Investigator.

Those subjects who had been exposed to any other investigational agent within 3 months prior to the enrolment to the study or who planned to travel (>4 days) to a region with high UVB exposure, or anyone with regular (>2/month) use of artificial UVB exposition, were excluded. Subjects on concomitant medication or drugs that may interfere with lipid absorption, products containing phosphorus, glycosides, magnesium-containing antacids, orlistat, thiazide diuretics, regular use of microsomal enzyme inducers (anticonvulsants, sedatives, etc.), corticosteroids (except for topical use), regular use of cholestyramine and other ion exchange resins, and laxatives were excluded. Pregnancy tests for all premenopausal women otherwise using oral contraceptive regimens were applied at screening, and also at the end of the trial, to exclude cases of pregnancy during the study treatment period. The dataset of those patients who had two or more follow-up lab tests missed during the treatment period was also excluded from our post hoc analysis.

There was no statistical difference in the main baseline parameters of age (*p*: 0.150), BMI (*p*: 0.501) serum calcium (*p*: 0.649), urine Ca/Cre (*p*: 0.422) between the groups of loading schedule and the active control low-maintenance dose patients, except a minimally higher (difference: 1.38 ng/mL) group mean in the initial of the baseline serum 25(OH)D level for the loading group patients, which was shown to be significant (*p*: 0.035). The patient distribution and baseline parameters of patients included in this post hoc safety analysis are summarized in Table 6.

Since there was essentially no difference in key baseline parameters between the patient groups of loading and the low-dose maintenance treatment arms, the two sets of safety data can be analyzed together to assess the frequency of adverse events of special interest. The nonparametric test of the three treatment groups confirmed the slight difference in baseline 25(OH)D between the two loading and the low-maintenance treatment arms (Kruskal–Wallis test *p*: 0.047). The loading efficacy will be discussed accordingly and separately in subgroup analysis.

The research protocols were approved by the Hungarian Medical Research Council’s Scientific and Ethical Committee (56215-0/2012-EKL; 27875-0/2018-EKL) and the national competent regulatory authority. All participants signed informed consent, and all the study-related procedures were conducted in accordance with ICH-GCP. The studies were registered at clinicaltrials.gov (NCT02069990; NCT04476511).

The methodology of laboratory of measurements was described earlier [12]. Briefly, the 25OHD and PTH were carried out by a direct, competitive chemiluminescence, fully-automated immunoassay (CLIA, LIAISON analyzer DiaSorin, MN, USA) and serum calcium, phosphate, alkaline phosphatase, potassium, sodium, lactate dehydrogenase, creatinine, urea, glucose, cholesterol, triglyceride, aspartate aminotransferase, alanine-aminotransferase, gamma-glutamyl transferase, urinary calcium, and phosphate were measured with a Beckman-Coulter automatic chemistry analyzer Au5800 (Beckman Coulter, Brea, CA, USA). Urinary calcium excretion was assessed as a ratio of urinary calcium-to-creatinine concentrations as described elsewhere [34].

Statistical methods for assessments of efficacy have been reported earlier [12,21]. The means ± standard deviations for continuous and frequencies for group categorical variables were used. For statistical tests, IBM SPSS Statistics for Windows, Version 28.0 (IBM Corp. Released 2021. IBM Corp. Armonk, NY, USA), and for non-parametric and regression models, the GraphPad Prism v.8.0.2 (GraphPad Software Inc., Boston, MA, USA) package was used to assess the equivalence in subgroup analysis. Individual changes in outliers were presented as value and the changes from UNL as a percentage. Descriptive statistics of changes are presented as means ± standard deviations unless otherwise specified.

## 5. Conclusions

The study conducted a comparative efficacy and safety evaluation of a rapid loading dosage of 300,000 IU vs. conservative low-dose supplementation and highlights the effectiveness of a loading–maintenance dosing schedule for correcting vitamin D deficiency.

The moderate–faster-loading regimen (300,000 IU) proved highly effective, with 100% of participants achieving normalization of vitamin D levels (above 30 ng/mL), regardless of BMI. In contrast, the slower-loading treatment showed less effectiveness for individuals with higher BMIs. Furthermore, low-dose maintenance therapy (1000 IU/day) failed to provide sufficient supplementation for overweight–obese patients, with only a few normal-weight individuals achieving target levels of 30 ng/mL after two months.

The findings suggest that BMI significantly influences the efficacy of vitamin D supplementation, indicating that overweight and obese individuals may need higher doses for optimal repletion. Age and gender were not significant predictors of loading efficacy, although they may affect maintenance outcomes, warranting further investigations.

Importantly, this rapid repletion strategy with a total loading dose of 300,000 IU did not increase the risk of hypercalcemia or hypercalciuria compared to the low-dose regimen. The study recommends a combination of the loading phase with a subsequent maintenance dose of 2000 IU daily to maintain adequate vitamin D levels.

## Figures and Tables

**Table 1:** The effectiveness of treatment was assessed by the changes in 25(OH)D levels in loading-dose groups vs. the low-dose maintenance group. The subgroup analysis by BMI is indicated in highlighted columns. (The statistically significant difference is marked with * (*p* < 0.05) or ** (*p* < 0.01) when groups or subgroups are compared).

	300,000 IU Loading							1000 IU/d Maintenance		
Change in 25(OH)D (ng/mL)	Loading Doses	Slower Loading 4286 IU Daily Equivalent			Moderate–Faster Loading 8571 IU Daily Equivalent			Low-Dose Maintenance 1000 IU Daily Equivalent		
	all pts.	s. load all pts.	BMI ≤ 25	BMI > 25	m. load all pts.	BMI ≤ 25	BMI > 25	all pts.	BMI ≤ 25	BMI > 25
Number of participants	70	35	17	18	35	14	21	62	22	40
Mean increase	28.06 **	25.08	30.39	20.06 **	31.04 *	34.34	28.51	11.44	15.28	9.31 **
Std. Deviation	11.93	10.16	10.25	7.24	12.95	15.55	10.99	6.15	6.19	5.08
Lower 95% CI of	25.21	21.59	25.12	16.46	26.59	24.94	23.51	9.88	12.46	7.68
Upper 95% CI of mean	30.91	28.57	35.67	23.66	35.49	43.74	33.51	13.00	18.09	10.93
Minimum	8.8	8.8	8.8	9.8	15.3	16.5	15.3	−0.55	2.7	−0.55
Median	25.9	23.5	31.8	18.8	27.9	33.6	27.3	6.6	15.4	9.7
Maximum	70.8	51.2	51.2	40.9	70.8	70.8	56.2	26.4	26.4	24.3
Range	62.0	42.4	42.4	31.1	55.5	54.3	40.9	26.9	23.7	24.8
5% Percentile	13.0	9.6	8.8	9.8	15.5	16.5	15.3	1.7	3.2	1.4
95% Percentile	50.0	47.7	51.2	40.9	59.1	70.8	55.0	25.1	26.4	18.5
Dose–response (ng/mL/100 IU/day)	-	0.585	0.709	0.468	0.362	0.406	0.333	1.145	1.528	0.931
Dose–response by Age ≤ 45 Age > 45	- -	0.612 0.553	- -	- -	0.347 0.368	- -	- -	1.137 1.147	- -	- -
Effectiveness rate (by BMI) rounded	-	28/35 (80%)	16/17 (94%)	12/18 (67%)	35/35 (100%)	100%	100%	6/62 (10%)	4/22 (18%)	2/40 (5%)
After 12 weeks of low-dose maintenance	-	-	-	-	-	-	-	20/62 (32%)	7/22 (32%)	13/40 (32%)
(Age ≤ 45)	-	9/11 (82%)	-	-	17/17 (100%)	-	-	4/19 (21%)	-	-
(Age > 45)	-	19/24 (79%)	-	-	18/18 (100%)	-	-	16/43 (37%)	-	-

Subgroup analyses based on age and gender revealed minor statistically nonsignificant (*p* = 0.255) effects on treatment outcomes. Gender differences were not fully explored due to the imbalance in the male-to-female ratio across treatment arms. Individual results were discussed in terms of outliers within each section of the safety analysis.

**Table 2:** Individual cases of supraoptimal (above 60 ng/mL) serum 25(OH)D as a result of loading treatment schedules. For safety assessment, serum calcium, phosphate, urine Ca/Cre, blood glucose, ALAT, ASAT results, and bone metabolic markers were added for each patient.

Patients	PAT#-317	PAT#-243	PAT#-716	PAT#-718
Treatment arm	slower loading	moderate–fast loading	moderate–fast loading	moderate–fast loading
Age	64	55	30	28
BMI	20.6	27.1	18.5	18.1
Gender	female	male	male	female
Season of treatment	fall	winter	winter	winter
Total loading dose applied	300,000 IU	300,000 IU	300,000 IU	300,000 IU
Treatment days (loading)	65	32	35	35
Daily dose equivalent as taken by the patients	4615 IU/d	9375 IU/d	8571 IU/d	8571 IU/d
Compliance	107.6%	109.4%	100%	100%
Concomitant medication	none	clopidogrel	naproxen	ibuprofen
		allopurinol	ibuprofen	dimenhidrinate
		atorvastatin	tramadol,	diclofenac
		nebivolol	paracetamol	topical fenazon + lidocain-HCl
Serum 25(OH)D (ng/mL)	Laboratory data			
Baseline	9.4	9.3	5.1	19.7
End of loading (300,000 IU)	60.6	65.5	75.9	68.7
Change by loading	+51.2	+56.2	+70.8	+49
Follow-up 4 weeks in maintenance (60,000 IU)	54.4	82.9	47.4	54.6
Safety and bone parameters				
Serum Ca after load (mmol/L)	2.27	2.27	2.45	2.52
Serum phosphate (mmol/L)	1.21	1.28	0.99	1.24
Urine CA/CRE ratio	0.02	0.31	0.44	0.36
Blood sugar (mmol/L)	5.2	5.4	5.3	5.0
SGOT (U/L)	21	24	17	23
SGPT (U/L)	20	10	16	14
GGT (U/L)	18	14	15	10
CTX (pg/mL) (baseline/load)	750/815	321/411	453/491	445/451
P1NP (ug/L) (baseline/load)	100.9/107.6	42.2/67.9	62.1/60.6	49.8/54.7
SAE reported	none	none	none	none
AE reported	none	none	none	-Palpitations, mild -Hematuria, mild -Ear pain, right, mild -Subfebrility, mild

**Table 3:** The safety assessment summary of the post-loading maintenance follow-up period for individual cases with the 25(OH)D > 60 ng/mL. For the safety assessment the serum calcium, phosphate and the urine CA/CRE data, blood sugar, and ALAT, ASAT, Gamma GT results by the end of maintenance vs. post-loading values were added for each patient. (The * indicated the values > UNL; “n.d.” = not done).

Patients	PAT#-206	PAT#-212	PAT#-243 *	PAT#-401	PAT#-813
Treatment arm	slower loading	moderate–fast loading	moderate–fast loading	moderate–fast loading	moderate–fast loading
Age	53	64	55	64	70
BMI	33.7	25.0	27.1	23.2	30.9
Gender	female	female	male	female	male
Season of treatment	winter	winter	winter	winter	spring
Total dose administered	360,000 IU	360,000 IU	360,000 IU	360,000 IU	360,000 IU
Treatment days (total)	102	60	53	70	63
Daily dose equivalent	3529 IU/d	6000 IU/d	6792 IU/d	5143 IU/d	5714 IU/d
Compliance	102.9%	116.7%	118.9%	100%	105%
Concomitant medication	piracetam nebivolol alprazolam losartan furosemide kalium metformin ASA spironolactone allopurinol rosuvastatin	dimethyl fumarate, diozmin+ herpesidin, pantoprazol vitamin B-complex, vitamin C	clopidogrel, allopurinol, atorvastatin, nebivolol,	euthyrox, cavinton forte, alprazolam, rosuvastatin	desloratadin, umeclidinium-bromide, salbuthamol
Serum 25(OH)D (ng/mL)	Laboratory data				
Baseline	12.3	12.0	9.3	10.7	17.5
End of loading (300,000 IU)	53.2	46.8	65.5	56.1	51.6
End of follow-up maintenance (60,000 IU)	62.4	84.5	82.9	82	61.9
Increase by the end of loading	+40.9	+34.8	+56.2	+45.4	+34.1
Change by post-loading maintenance	+9.2	+37.7	+17.4	+25.9	+10.3
Serum Ca (mmol/L) Load/post-load maintenance	2.5/2.44	2.39/2.41	2.27/2.37	2.3/2.25	2.52/2.38
Serum Phosphate (mmol/L) Load/post-load maintenance	1.34/1.45	1.16/1.08	1.21/1.06	1.2/1.26	n.d./n.d.
Urine CA/CRE ratio Load/post-load maintenance	0.92 */0.53	0.18/n.d.	0.02/0.02	0.72 */1.16 *	0.17/0.51
Blood sugar (mmol/L) Load/post-load maintenance	5.1/5.5	4.2/5.9	5.4/4.9	5.4/5.2	4.8/4.8
SGOT (U/L) Load/post-load maintenance	15/19	23/20	24/26	21/23	23/18
SGPT (U/L) Load/post-load maintenance	12/16	24/25	20/20	13/16	19/13
GGT (U/L) Load/post-load maintenance	13/14	16/16	14/13	14/13	29/27
SAE reported	none	none	none	none	none
AE reported	none	none	none	Oxalate in urine mild/moderate, recovered	none

**Table 4:** Assessment of effectiveness by BMI subcategories.

Patients/Subgroups	All Deficiency 25OHD < 20 ng/mL	Deficiency with BMI ≤ 25	Deficiency with BMI > 25
Percent of normalization	Proportion (%)	Proportion (%)	Proportion (%)
Moderate–faster loading of 300,000 IU	100%	100%	100%
Slower loading of 300,000 IU	79%	94%	67%
1000 IU/d maintenance	10%	20%	0%

**Table 5:** Summary of dosing strategies of cholecalciferol in loading and in low-dose maintenance studies.

Supplementation	Moderate–Faster Loading (“m.load”)	Slower Loading (“s.load”)	Low Dose Maintenance (“l.maint”)
**Loading phase**			
Loading dose applied	60,000 IU * /week	30,000 IU /week	30,000 IU /month
Duration	5 weeks	10 weeks	2 months
Total dose of loading	300,000 IU (7500 μg)	300,000 IU (7500 μg)	~60,000 IU (1500 μg)
Dose equivalent	8571 IU/d (214.3 μg/d)	4286 IU/d (107.15 μg/d)	1000 IU/d (25 μg/d)
**Follow-up maintenance phase**			
Maintenance dose applied	30,000 IU /2 weeks	30,000 IU /2 weeks	1000 IU /day
Duration	1 month	1 month	1 month
Total dose	60,000 IU (1500 μg)	60,000 IU (1500 μg)	~30,000 IU (750 μg)
Dose equivalent	2000 IU/d (50 μg/d)	2000 IU/d (50 μg/d)	1000 IU/d (25 μg/d)
Total dose applied	360,000 IU (9000 μg)	360,000 IU (9000 μg)	~90,000 IU (2250 μg)

* Applied in two 30,000 IU doses apart 2–3 days a week.

**Table 6:** Baseline characteristics of patients in loading-dose and low-dose maintenance groups. The subcategories for subgroup analysis (age, BMI) are highlighted under the main parameters, respectively.

Main Group Characteristics at Baseline	300,000 IU Slower Loading Arm		300,000 IU Mod-Fast Loading Arm		Sign. (*p*)	All Loading Dose Arms		All 1000 IU/d Low-Dose Maint.		Sign. (*p*)
	n	Eq:4286 IU/d	n	Eq:8571 IU/d		n	300,000 IU	n	56,000 IU	
Male/Female		5/30		12/23			17/53		12/50	
**Age (years)**	35	51.2 ± 14.9	35	47.2 ± 14.5	n.s.	70	49.2 ± 14.7	62	53.1 ± 16.0	n.s.
Subcategories Age ≤ 45	11	32.1 ± 6.5	17	34.4 ± 7.7		28	33.5 ± 7.2	19	32.5 ± 7.2	n.s.
Age > 45	24	60.0 ± 7.2	18	59.3 ± 6.5		42	59.7 ± 6.8	43	61.9 ± 8.6	n.s.
**Body mass index** **(BMI; kg/m^2^)**	35	26.41 ± 5.30	34	26.31 ± 4.88	n.s.	69	26.36 ± 5.06	62	27.02 ± 4.97	n.s.
Subcategories BMI ≤ 25	17	21.68 ± 2.19	13	21.38 ± 2.46		30	21.57 ± 2.37	22	21.98 ± 2.00	n.s.
BMI > 25	18	30.85 ± 2.92	21	29.39 ± 3.04		39	30.05 ± 3.07	40	29.79 ± 3.77	n.s.
**25(OH)D (ng/mL)**	35	14.15 ± 4.00	35	15.01 ± 3.84	n.s.	70	14.58 ± 3.91	62	13.20 ± 3.90	0.035
**Serum calcium (nmol/L)**	34	2.41 ± 0.12	34	2.38 ± 0.11	n.s.	68	2.40 ± 0.11	62	2.39 ± 0.09	n.s.
**Urinary** **calcium/creatinine**	31	0.3 ± 0.3	30	0.3 ± 0.2	n.s.	61	0.3 ± 0.2	62	0.33 ± 0.26	n.s.

n.s. = not significant.

## Data Availability

The complete set of research data is available only upon request to Sponsor Pharma Patent Ltd. concerning the “PAT12-730DS” and “PAT17-LOADS studies due to data exclusivity of Sponsor.

## Appendix A

Figure A1: Changes in serum calcium during the post-loading maintenance dosing period.
